# Selective amplification of *Brucella melitensis *mRNA from a mixed host-pathogen total RNA

**DOI:** 10.1186/1756-0500-3-244

**Published:** 2010-09-28

**Authors:** Carlos A Rossetti, Cristi L Galindo, Harold R Garner, L Garry Adams

**Affiliations:** 1 Department of Veterinary Pathobiology, College of Veterinary Medicine, Texas A&M University, College Station, TX, USA; 2 Department of Biochemistry and Internal Medicine, University of Texas Southwestern Medical School, Dallas, TX, USA; 3 Instituto de Patobiología, CICVyA-CNIA, INTA. CC25 (B1712WAA) Castelar, Buenos Aires, Argentina; 4 Virginia Bioinformatics Institute, Virginia Polytechnic and State University, Blacksburg, VA, 24060, USA

## Abstract

**Background:**

Brucellosis is a worldwide anthropozoonotic disease caused by an in vivo intracellular pathogen belonging to genus *Brucella*. The characterization of brucelae transcriptome's during host-pathogen interaction has been limited due to the difficulty of obtaining an adequate quantity of good quality eukaryotic RNA-free pathogen RNA for downstream applications.

**Findings:**

Here, we describe a combined protocol to prepare RNA from intracellular *B. melitensis *in a quantity and quality suitable for pathogen gene expression analysis. Initially, *B. melitensis *total RNA was enriched from a host:pathogen mixed RNA sample by reducing the eukaryotic RNA..Then, to increase the *Brucella *RNA concentration and simultaneously minimize the contaminated host RNA in the mixed sample, a specific primer set designed to anneal to all *B. melitensis *ORF allows the selective linear amplification of sense-strand prokaryotic transcripts in a previously enriched RNA sample.

**Conclusion:**

The novelty of the method we present here allows analysis of the gene expression profile of *B. melitensis *when limited amounts of pathogen RNA are present, and is potentially applicable to both *in vivo *and *in vitro *models of infection, even at early infection time points.

## Background

Brucellosis is a worldwide anthropozoonotic disease caused by small intracellular Gram negative coccobacilli belonging to genus *Brucella*. In spite of the massively underreported cases, brucellosis is considered the world's most widespread zoonotic infection [1]. Basically, human brucellosis is an occupational-related disease associated with accidental contact with infected animals or clinical specimens, inhalation of infected aerosolized particles, or a food-borne disease associated with the consumption of contaminated animal products [2]. Clinically, human brucellosis is an incapacitating disease which presents a spectrum of severity and symptoms varying dependent on the species of *Brucella*. *B. melitensis *causes the most severe and acute symptoms in humans such as intermittent fever, chills, sweats, weakness, myalgia, osteoarthricular complications, endocarditis, depression and anorexia, frequently resulting in chronic debilitating illness [3].

To date, little is known about *Brucella *gene expression during host:pathogen interaction. The published studies have relied on the use of *gfp *reporter gene system [4] or transposon mutagenesis [5] rather than direct analysis of *Brucella *transcripts. The characterization of the transcriptome of intracellular pathogens during host-pathogen interaction has been limited due to the difficulty of obtaining an adequate quantity of good quality eukaryotic RNA-free pathogen RNA for downstream applications. Basically, until now few experimental approaches were designed to minimize the presence of eukaryotic RNA and increase the relative amount of pathogen RNA collected from infected cells. One approach is to lyse infected host cells with an ice-cold mixture of acid phenol, ethanol and detergent, followed by collection by centrifugation and RNA extraction using a standard RNA purification procedure [6]. Gene expression profiles of intramacrophage *Salmonella typhimurium*, *Listeria monocytogenes *and *Shigella flexneri *were studied using this methodology [6-8]. Another successful approach developed entails lysing host cells with cold water, followed by enzymatic digestion of host genetic material with RNase and DNase while bacteria remain protected by their cell envelope [9]. A different approach consists of isolation of total RNA from infected cells, followed by removal of host mRNA using oligo(dT) columns or magnetic beads, or by greatly reducing host RNA through selective reverse transcription of bacterial mRNA. The intracellular transcriptional profile of *Chlamydia trachomatis *during HeLa cells infection, *Burkholderia pseudomallei *during acute melioidosis in the hamster model of infection and *Mycobacterium tuberculosis *in infected mice were analyzed by this procedure [10-12]. However, further studies are limited by all these methodologies if small amounts of pathogen mRNA is recovered, such as during early infection time points. To have a sufficient template for downstream applications, one possibility might be to increase the concentration of the inoculum or the samples, i.e. more animals or tissues inoculated, which it is not always possible. Another alternative would be to amplify the pathogen mRNA isolated from a mixed sample. Few methods to amplify signals from bacteria transcripts have been reported. One method, Differential Expression using Customized Amplification Libraries (DECAL) [13], has not been demonstrated to work with *in vivo *material. The method described by Motley *et al*. (2004) produced antisense RNA which could not be used to hybridize on the oligo-arrays [14], and like the Linear Amplification of Prokaryotic Transcripts (LAPT) [15], does not discriminate RNA populations during amplification.

We have developed a combined protocol to prepare RNA from intracellular *B. melitensis *in a quantity and quality suitable for pathogen gene expression analysis. First, *B. melitensis *RNA was enriched from a host:pathogen mixed RNA sample, and then, the prokaryotic transcripts were selectively amplified. The method we present here allows analysis of the gene expression profile of intracellular *B. melitensis*, even at early infection time points when few numbers of pathogens are present.

## Materials and methods

### Bacterial strains, media and culture conditions

Smooth virulent *Brucella melitensis *16 M Biotype 1 (ATCC 23456) (American Type Culture Collection, Manassas, VA), re-isolated from an aborted goat fetus was maintained as frozen glycerol stocks. Saturated cultures were sub-cultured into tryptic soy broth (TSB) (BD, Franklin Lakes, NJ) and incubated for 18 h with shaking (200 rpm) at 37°C with 5% CO_2 _until the log growth phase (OD_600 _= 0.9) was reached.

### Isolation of total RNA and genomic DNA from *B. melitensis *16 M culture

*B. melitensis *total RNA and genomic DNA was isolated as previously described [16]. Briefly for RNA isolation, ice-cold ethanol/phenol solution was added to the *B. melitensis *culture at late-log growth phase and the bacteria recovered by centrifugation. The media was then removed, and the pellet suspended in TE buffer-lysozyme solution containing 10% SDS (Ambion). After 2 min of incubation, acid water-saturated phenol (Ambion) was added to the lysate and mixed, and the sample incubated for 6 min at 64°C. Tubes were kept on ice for at least 2 min and then centrifuged at maximum speed. The upper layer, containing the RNA, was transferred to a new tube, mixed with an equal volume of chloroform (Sigma) and then separated by centrifugation. The aqueous phase was mixed with 100% cold ethanol and stored at -20°C. After at least one hour of incubation, RNA was pelleted by centrifugation, washed in 80% ethanol and suspended in DEPC-treated water (Ambion) with 2% (w/v) DTT and 1% (w/v) RNase inhibitor (Promega). Contaminant genomic DNA was removed by RNase-free DNase I treatment (Ambion) according to the manufacturer's instructions, and samples were stored at -80°C until used. RNA concentration was quantified by NanoDrop^® ^ND-1000 (NanoDrop), and the RNA quality was assessed using the Agilent 2100 Bioanalyzer (Agilent).

For isolation of *B. melitensis *gDNA, a pellet from a saturated culture of *B. melitensis *16 M grown in tryptic soy broth (TSB) (BD) was washed with 25 ml of J-buffer [0.1 M Tris pH 8.0; 0.1 M EDTA; 0.15 M NaCl] and then lysed in 1 ml of J-buffer containing freshly made 10% lysozyme solution [10 mg/ml in 0.25 M Tris, pH 8.0] at 37°C for 10 min. Later, 0.1 ml of RNAse solution (1 mg/ml) was added and incubated at 37°C for another 10 min. Finally, the solution was heated to 70°C for 3 min. DNA was released from the cells by adding 0.08 ml of 30% sodium salt of N-lauroyl sarcosine (Sigma) and incubated at 37°C for 1 h, followed by digestion of proteins by 4 mg of proteinase K. The resulting solution was dialyzed against TE [10 mM Tris, pH 8.0 and 1 mM EDTA] overnight at 37°C. Next day, the preparation was transfered back to a plastic tube and DNA was subsequently twice extracted with ten minute of gentle inversion in neutral, water-saturated phenol, followed by two-time ether extract. Finally, DNA was again dialyzed for 8 h against TE. DNA concentration was quantified by NanoDrop^® ^ND-1000 (NanoDrop) and stored at 4°C until used.

### Isolation of total RNA from HeLa and MDBK cell lines

Total RNA from HeLa S3 and Madin-Darby bovine kidney (MDBK) (ATCC) cell cultures was extracted by TRI-Reagent^® ^(Ambion, Austin, TX) according to manufacturer's instructions. The resultant RNA pellet was re-suspended in DEPC-treated water (Ambion) with 2% DTT and 1% RNase inhibitor (Promega, Madison, WI). Contaminant genomic DNA was removed by RNase-free DNase I treatment (Ambion) according to the manufacturer's instructions, and samples were stored at -80°C until used. RNA concentration was quantified by NanoDrop^® ^ND-1000 (NanoDrop, Wilmington, DE), and the RNA quality was determined using the Agilent 2100 Bioanalyzer (Agilent, Palo Alto, CA).

### Design of *B. melitensis *genome-directed primers (*Bm*GDPs)

GDPs were designed using GDP-Finder, a computer-based algorithm that predicts the minimal number of primers to specifically anneal to all ORFs in a given genome [17]. Searching for the first 500 bp of each ORF complementary sequence, the algorithm predicted that 89 different reverse primers of 8-mer oligonucleotides were required to anneal to the 3,198 *B. melitensis *ORFs (Additional file 1). Primers were commercially synthesized (Sigma Genosys, The Woodland, TX) and used for reverse transcription during the first step of RNA amplification and for labeling the cDNA.

### Enrichment and sense-strand amplification of *B. melitensis *mRNA from mixed host-pathogen total RNA sample

*B. melitensis *total RNA was initially enriched from two spiked samples of 25 μg of total RNA extracted from HeLa or MDBK cells culture. The enrichment procedure was performed using the MICROB *Enrich*^® ^kit (Ambion) according to the manufacturer's instructions. After enrichment, the remaining RNA was precipitated in 100% ethanol at -20°C for at least 1 h, centrifuged for 30 min at 10,000 × *g *at 4°C, and washed twice in ice-cold 70% ethanol. After 5 min centrifugation at 10,000 × *g *at 4°C, the RNA was re-suspended in 25 μl of DEPC-treated water (Ambion) and immediately amplified in a 3 step-protocol, previously described in detail [15]. Briefly, the total amount of RNA after the enrichment procedure was reverse transcribed to cDNA in a 50 μl reaction using 42 μM of *Bm*GDPs, 5 μl of 50× dNTPs (10 mM each) (Invitrogen, Carlsbad, CA), 2.5 μl of PowerScript (Clontech, Palo Alto, CA), 2.5 μl of RNAsin (Promega), and 42 μM of T7 promoter-template switching primer (T7-TS) (5'-CGAAATTAATACGACTCACTATAGGGAGAGTACGCGGG-3') (Sigma Genosys). The *Bm*GDPs and the RNA were mixed and heated at 70°C for 10 min before being placed on ice for >3 min and addition of the T7-TS and reverse transcription reagents. The first-strand and template switching reaction were performed at 42°C for 90 min in a thermocycler with a non-heated lid (DNA Engine, MJ Research, Inc., Waltham, MA). In the next step, the second-strand cDNA was synthesized by adding 1× final concentration of 10× Advantage 2 Polymerase buffer (Clontech), 1× final concentration of 50× dNTPs mix (10 mM each) (Invitrogen), 2U of Rnase H (Roche, Indianapolis, IN) and 1× final concentration of 50× Advantage 2 Polymerase mix (Clontech) to the final reaction volume of 150 μl. The components were mixed and the reaction incubated in a heated-lid thermocycler (DNA Engine, MJ Research, Inc.) with the following cycle: 37°C for 5 min, 94°C for 2 min, 65°C for 1 min and 75°C for 60 min. Double-stranded cDNA was purified using PCR purification kit (Qiagen, Valencia, CA), eluted in 100 μl of nuclease-free water and concentrated to 15 μl in a speed-vac with no heat. In the last step, the *in vitro *transcription, using the double-stranded cDNA as the template and T7 Megascript kit (Ambion) in 40 μl reactions with an additional 400U of T7 polymerase (Ambion) and 20U of Rnase inhibitor SUPERase-In (Ambion), was performed at 37°C for 16 h. RNA was cleaned and recovered using RNeasy kit (Qiagen) and eluted in 100 μl of nuclease-free water with 40U of SUPERase-In.

### Construction of *B. melitensis *cDNA microarrays

Microarrays containing all *B. melitensis *16 M ORFs were designed at the Pathogen Expression Core (Dr. S.A. Johnston's Laboratory at Arizona State University) as previously explained [16].

### Sample preparation and slide hybridization

The labeling and hybridization procedures was taken from the protocol developed by The Institute for Genomic Research [18] and adapted for our experiments [16]. Briefly, 10 μg of total RNA from *B. melitensis *16 M were reverse transcribed overnight to amino-allyl cDNA using 1.5 μg of *Bm*GDPs or 6 μg of random hexamer primers (Invitrogen). The reaction was stopped by incubating the samples with 1 M NaOH and neutralized with 1 M HCl. Samples were incubated with Cy3 dye ester (Amersham Pharmacia Biosciences, Piscataway, NJ) in 0.1 M sodium carbonate buffer (pH 9,0) in the dark for one hour. For labeling of *B. melitensis *gDNA, 1.5 μg of isolated DNA was directly labeled overnight using Bioprime DNA labeling system (Gibco) according to the manufacturer's instructions, with the following modifications: 63 μM of *Bm*GDPs was used instead of random hexamer primers, and 5 μl of 10× dNTPs mix [1.2 mM each dATP, dGTP, dTTP; 0.6 mMdCTP; 10 mM Tris pH8.0; 1 mM EDTA] (Invitrogen) was used instead of the mix from the kit along with 3 μl of Cy5dCTP (1 mM stock; Amersham). In both cases, uncoupled dye was removed using PCR purification kit (Qiagen) and dye incorporation calculated by NanoDrop^® ^ND-1000 (NanoDrop).

Dried, labeled cDNA samples were resuspended in nuclease-free water (Ambion) and mixed with 0.5 μg of labeled *B. melitensis *gDNA to the final volume of 35 μl. Samples were heated at 95°C for 5 min and then kept at 45°C until hybridization. Thirty five μl of 2× formamide-based hybridization buffer [50% formamide; 10× SSC; 0.2% SDS] was added to each pre-annealed samples, well mixed and applied to a pre-treated custom 3.2 K *B. melitensis *oligo-array. Slides were hybridized at 45°C for ~20 h in a dark, humid chamber (Corning) and washed for 10 min at 45°C with low stringency buffer [1× SSC, 0.2% SDS] followed by two 5-min washes in a higher stringency buffer [0.1× SSC, 0.2% SDS and 0.1× SSC] at room temperature with agitation. Slides were dried by centrifugation at 800 × *g *for 2 min and immediately scanned.

### Data acquisition and microarray data analysis

Microarrays were scanned using a commercial laser scanner (GenePix 4100; Axon Instruments Inc., Foster City, CA) with independent excitation of the fluorophores Cy3 and Cy5. Fluorescent signal and local background intensities were quantified for each spot by using image analysis software (GenePixPro 6.0; Axon Instruments Inc.). Genes with fluorescent signal values below background were disregarded in all analyses and the arrays were normalized against *B. melitensis *genomic DNA, as previously described [19]. Data were analyzed using GeneSifter (VizX Labs, Seattle, WA). Regression analysis, Pearson's correlation coefficient and Spearman's rank correlation were used to assess inter-array and intra-array variability. After background subtraction and normalization, the signal values of every gene (triplicate spots in 2 arrays = 6 spots) for each experiment were averaged and pair-wise comparisons performed for each group of samples. Individual pair-wise comparison was performed to ensure reproducibility across replicates and for elimination of transcript differences caused by normal sample variation. Student's *t *test was also performed, and genes were deemed as significant if the *p *value was ≤ 0.05.

## Results

### Genome-directed primers (GDPs) generate more specific probes of *B. melitensis *transcripts than random hexamer primers (RHPs)

**Figure 1:**
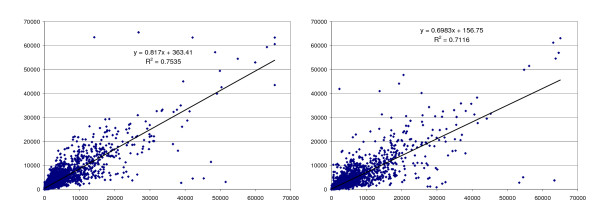
**Inter-array comparison of signal consistency from *B. melitensis *RNA samples reverse transcribed with Genome Directed Primers (GDPs) vs. Random Hexamer Primers (RHPs)**. The consistency of the signal generated from a Cy3-labeled *B. melitensis *cDNA using a primer sets predicted from GDP-Finder software (GDPs) was compared with a Cy3-labeled *B. melitensis *cDNA made by a commercially available set of RHPs. Each method was used on two sets of identical *B*. *melitensis *RNA samples isolated from late-log phase cultures, and the resulting cDNA was labeled with Cy3 and co-hybridized with Cy5-labeled genomic DNA (gDNA) to two *B. melitensis *oligoarrays for each primer type. Signal consistency from samples reverse transcribed with GDPs was slightly higher (R^2 ^= 0.7535) (**A**) compared to RHPs samples (0.7116) (**B**).

Talaat *et al*. (2004) and Lawson et al. (2006) demonstrated the potential usefulness of GDPs for the detection of *Mycobacterium tuberculosis *and *Yersinia pestis *gene expression *in vivo*, respectively [11,20]. In a technical experiment designed to determine the usefulness of the GDPs for generation of cDNA from *B. melitensis *mRNA and subsequent hybridization to microarrays, a primer set (*Bm*GDPs) generated from GDP-Finder software was compared with a commercially available set of RHPs. Each method was used on two identical *B*. *melitensis *RNA samples isolated from log phase cultures, and the resulting cDNA was labeled with Cy3 and co-hybridized with Cy5-labeled genomic DNA (gDNA) on two arrays for each primer type. Initially, the amount of cDNA generated was lower using *Bm*GDPs than RHPs (5.7 μg vs. 7.5 μg from every 10 μg of total RNA), which could be due to the selective annealing of *Bm*GDPs to *B. melitensis *mRNA. For inter-array comparison after labeling and hybridization, 63 of 9,681 spots with signal values flagged "bad" by GenePixPro 6.0 were removed across all four data sets to make them comparable. The consistency of the signal from samples reverse-transcribed with GDPs was slightly higher (R^2 ^= 0.7535) compared to RHPs samples (R^2 ^= 0.7116) (Figs. 1A and 1B). Linear regression analysis also revealed a slightly higher advantage for GDPs (*P *value of 1.12 × 10^-72^, *T *statistic of 18.2) over RHPs (*P *value of 1.0 × 10^-14^, *T *statistic of 7.7). Likewise, correlation analysis indicated that GDPs (87%) were slightly more consistent than RHPs (84%), and the consistency was greater when considering average signal intensity values (GDPs = 1665 & 1724 vs. 1912 & 1492 of RHPs), average standard deviation (490 vs. 540) and standard deviation of average intensities (41 vs. 297). We also compared the average ratio between experimental replicates (1.3 for GDPs and 0.91 for RHPs), as well as the average signal log ratio between each experimental replicate and the co-hybridized gDNA (0.77 and 0.69 for GDPs and 0.69 and 0.83 for RHPs). While the average ratio between experimental samples was higher for GDPs than for RHPs, the standard deviation of signal log ratios, which is a more reliable measure of consistency between arrays, was lower (0.06 for GDPs and 0.10 for RHPs). These results indicate greater consistency between arrays hybridized with samples reverse-transcribed using GDPs rather than RHPs.

We next examined the consistency between replicate spots on each array (intra-array comparison). To streamline the comparison, genes that were flagged as "bad" and any "matching" replicate spots were removed across all arrays. Eighty-one spots were eliminated, which left 9,600 spots representing 3,200 different genes (genes spotted in triplicate). The comparison of the average signal values (GDPs = 1,668 & 1,726, RHPs = 1,915 & 1,494), their standard deviations (42 v 298) and the average standard deviation between replicate spots (GDPs = 257 & 190, RHPs = 296 & 290) essentially confirmed a higher intra-array consistency for the GDPs over the RHPs samples. As a final measure of consistency/variability between arrays, we separated all of the replicate spots and treated them as replicate samples/arrays in order to gauge the trend of consistency across all replicate spots: two replicates (arrays) with triplicate spots for each, yields 6 "theoretical arrays" for each condition (GDPs and RHPs). The average R^2 ^value was much higher for GDPs (0.83) compared to RHPs (0.74), despite similar standard deviations (0.10 for GDPs and 0.09 for RHPs). These results indicated that GDP replicate spots were more similar to one another than were RHP replicate spots. Altogether, these results indicate that the reverse transcription of *B. melitensis *RNA using GDPs generates more specific probes than those generated with RHPs.

### *Bm*GDPs and probes printed on the pathogen array select against the hybridization of eukaryotic transcript

As *Bm*GDPs are 8-mer oligonucleotide long primers, a potential concern that they anneal and amplify contaminating RNA sequences from the host that overlap with sequences of the *B. melitensis *transcripts and cross-hybridized with probes on *B. melitensis *oligoarrays, was considered. Total RNA isolated from HeLa cells culture was labeled and hybridized on *B. melitensis *microarrays under the same conditions as RNA isolated from *Brucella *cultures. Initially, we observed a reduced amount of cDNA generated (3.8 μg vs. 9 μg from 10 μg of HeLa RNA, and 3.1 μg vs. 8.8 μg from 10 μg of MDBK RNA using *Bm*GDPs or RHPs, respectively; vs. 5.7 μg from 10 μg of *B. melitensis *RNA), which indicated some restriction of priming of *Bm*GDPs to eukaryotic RNA. When the cDNA generated from host RNA using *Bm*GDPs was hybridized on the pathogen array, only 105 of 3120 genes (3.3%) were consistently detected (raw hybridization signal intensity values above 500 for all 6 replicates -triplicate arrays on duplicate slides-), vs. 34% when RNA from *B. melitensis *was used. These data illustrate that *Bm*GDPs biased priming to *B. melitensis *transcripts, along with the oligonucleotides printed on pathogen array, highly reduce the chances of eukaryotic transcripts to hybridize on the array.

### The enrichment procedure enhanced *B. melitensis *RNA concentration from the mixed *Brucella*:host RNA sample

**Figure 2:**
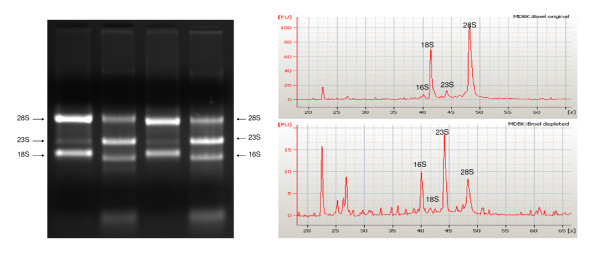
**Integrity and composition of the host:*Brucella *RNA samples pre- and post-treatment**. Twenty-five (25) μg total eukaryote RNA from human (HeLa S3) and bovine (MDBK) cell lines were mixed with 2 μg of *Brucella melitensis *16 M RNA (ratio 12.5:1) and treated with MICROB *Enrich*™(Ambion) according to the instruction manual. **(A) **Agarose gel electrophoresis image. Lines 1 & 2: HeLa cell line:*B.melitensis *16 M RNA mix; lines 3 & 4: MDBK cell line:*B.melitensis *16 M RNA mix; pre and post-treatment respectively. **(B) **Comparison of RNA composition from a sample of MDBK cell line:*B. melitensis *16 M pre and post-treatment with MICROB *Enrich*™ (Ambion) and examined on an Agilent 2100 Bioanalyzer. Ribosomal RNA subunits are indicated.

To minimize the chances of falsely detected pathogen genes due to the presence of eukaryotic RNA, we enriched *Brucella *RNA from a host:pathogen mixed RNA sample before amplification. Twenty-five μg of total eukaryote RNA from HeLa or MDBK cell lines was mixed with 2 μg of *B. melitensis *RNA (ratio 12.5:1). The MICROB *Enrich*^® ^kit (Ambion) was then used to remove the eukaryotic RNA from the mixed sample. The total RNA yield after treatment was 3.72 μg (an 86.2% reduction) and 4 μg (85% reduction) for HeLa:*Brucella *and MDBK:*Brucella *mix, respectively. The integrity and composition of the samples pre- and post-treatment were evaluated by agarose gel electrophoresis and bioanalyzed (Fig. 2A and 2B). These data effectively demonstrate the usefulness of the enrichment procedure to enrich the pathogen RNA and reduce the chances that undesirable RNAs (i.e. host RNA) were inadvertently subsequently amplified and eventually hybridized on the pathogen array.

### The biological information of *B. melitensis *transcriptome is conserved after enrichment and amplification of mixed RNA sample

To increase the template concentration when small amounts of pathogen mRNA are present in the sample, such as the early stage of infection, we linearly amplified the enriched *Brucella *RNA. An important consideration to take in account when extra procedures are applied on the original samples is how well the fidelity of the original information is maintained. A degree of potential bias that may be introduced using the enrichment and amplification (E&A) procedures and the measure of the reproducibility of the protocol were evaluated by spiking 2 different samples of 25 μg of total RNA extracted from HeLa or MDBK cell culture with 0.2 μg of *B. melitensis *RNA (host:pathogen RNA = 125:1). The mixed RNA samples were initially enriched, which decreased the RNA concentration more than 88% (from 25 μg to less than 3 μg of total RNA). Then, 1:10 volume of the remaining RNA (i.e. 0.25 and 0.3 μg of total RNA) was amplified using a 3 step protocol described in the M&M. This protocol enables amplification of sense-stranded prokaryotic transcripts. One round of amplification yielded more than 80 μg of amplified sense total RNA from each sample (>than 260-fold amplification). Two aliquots of 10 μg from every E&A RNA sample were indirectly labeled and co-hybridized against *B. melitensis *gDNA on *B. melitensis *microarrays (4 arrays). The degree of bias introduced by the enrichment and amplification was determined by evaluating the performance of the E&A RNA samples against identical labeling and hybridization protocol from non-treated *B. melitensis *RNA (i.e. RNA extracted from a log culture).

Prior to analysis, spots that were flagged as "unacceptable" were removed leaving a total of 3,120 acceptable spots (out of the original 3,227). We employed genomic normalization to improve data quality and to compare multiple samples in a minimum of experiments [19]. To compare the consistency of gene expression for each sample type, the signal values for each slide were graphed pairwise and fitted with trend lines. The resulting R^2 ^values (slopes) represented the level of consistency (similarity) between each slide, which was similar for biological and technical replicates (Table 1). The overall level of consistency for controls was less than for experimental replicates between slides. Also inter-slide variability was higher than intra-slide variability. E&A treatment decreased this variability, which suggests that some level of signal for the *B. melitensis *control slides might have been due to noise interference. We next examined the average signal values for *B. melitensis *control RNA and for E&A mixed RNA. The results revealed higher signals in control samples (987.5 vs. 800.5), but the difference was small and indicates that amplification does not necessarily correspond to a higher signal.

**Table 1:** Results of pairwise graphical comparisons

Samples	**R**^**2 **^**value**	
	*Control (Cy5)*	*Experimental*
*Slides (inter)*		
*B. melitensis *control	0.49	0.58
HeLa:*Bmel *E&A	0.52	0.79
*Arrays (intra)*		
*B. melitensis *control	0.88	0.86
HeLa:*Bmel *E&A	0.86	0.97

E&A: enriched and amplified

In order to evaluate the bias introduced by the enrichment and amplification process and the reproducibility of the protocol, we first calculated the correlation (Spearman's rank correlation coefficient, *r*) between the fluorescence intensity in E&A vs. control samples and then compared the expression profiles of hybridizations of 2 independently amplified RNA samples. Our results confirmed a good correlation (*r *= 0.8496) between the expression profile of the E&A samples against the control sample and a high degree of correlation between independent replicates (*r *= 0.9823). Altogether, these results suggest that our protocol is highly reproducible with a high level of maintenance of the original information. Considering the fact that the magnitude of the bias can be under-estimated when it is highly reproducible [21], our results from preliminary experiments indicate that enrichment of the original RNA sample followed by the biased amplification of pathogen transcripts is sufficient to accurately characterize the transcriptome of intracellular pathogens in *in vitro *or *in vivo *systems of infection.

## Discussion

Modern technology has made gene expression detection of mammalian systems straightforward and robust. However, the study of the transcriptional profile of intracellular bacteria is challenging due to the difficulty of obtaining adequate high quality pathogen RNA free of eukaryotic-RNA for downstream applications. To reduce the interference of host RNA from the heterogeneous population of RNA, we used a commercial kit to significantly reduce the eukaryotic RNA and enrich the *Brucella *RNA. However, the enrichment procedure by itself does not address the challenge of a small amount of *Brucella *RNA present in the initial material (i.e., at the onset of the infection) that is insufficient for microarray studies. To increase the *Brucella *RNA concentration and simultaneously minimize the contaminated host RNA in the mixed sample, we applied a protocol of linear amplification of sense-stranded RNA biased to pathogen transcripts in the previously enriched RNA sample. In the first step, the protocol utilizes *Bm*GDPs to bias the reverse transcription to bacterial transcripts and the overhang tailing activity of Moloney murine leukemia virus (MMLV) reverse transcriptase to add the T7 promoter to cDNAs during reverse transcription. Then, the second-strand cDNA is synthesized, and finally *in vitro *transcription is carried out using a T7 polymerase. The methodology was reproducible, as indicated by correlation analysis of the gene expression detected in an original sample (non-enriched non-amplified *B. melitensis *RNA) vs. an E&A host:pathogen mixed RNA sample (i.e. *B. melitensis *RNA spiked with HeLa RNA = 1:125). Bearing in mind that the presence of contaminant eukaryotic RNA and the extra handling of the samples could introduce some degree of bias into the population of treated RNA [22], the analysis of our results yielded a high correlation (*r *= 0.8496) between these 2 samples with high reproducibility of the technique (correlation between independent replicates = 0.9823). This result is in concordance with previous correlation study of pathogen gene expression from a mixed host:pathogen RNA sample with the original pathogen RNA sample [23]. Other studies focused on validation of the methodology for RNA amplification in the sense orientation showed a correlation between "pure" samples before and after amplification. Making the same comparison (i.e. unamplified vs. amplified *B. melitensis *RNA), our study yielded a higher correlation level compared to other studies [*r *= 0.81398 vs. 0.77 [15] or 0.8009 [22]) (data not shown). Amplification of mixed RNA samples without previous enrichment did yield the lowest correlation compared to the original *B. melitensis *RNA sample (*r *= 0.38296). These results differ from another study that found a more complete bacteria global expression profile applying direct amplification on the host:pathogen mixed sample [20]. A possible explanation may be that the ratio of bacterial:host RNA in our study was much lower, as a consequence of the presence of a higher level of contaminant eukaryotic RNA.

Another potential concern was that eukaryotic RNA present in the sample may interfere with pathogen gene expression detection. This possibility is remote due to the different levels of filters applied (i.e., the initial enrichment treatment of the sample, the bias primed of *Bm*GDPs to *B. melitensis *transcripts, the specific sequences of probes on the pathogen array), which select against the host RNA amplification, labeling and hybridization. However, if one desires to eliminate any doubt, one could hybridize in parallel, the untreated infected sample on the pathogen array and disregard from future analysis all those spots that produced a signal in the untreated sample. This novel alternative, which has not been reported in previous studies that have evaluated the *in vivo *or *in vitro *transcriptional profile of intracellular bacteria using similar strategies [11,12,24], decreases the sensitivity but increases the specificity of the system (i.e. some true positive expressed pathogen genes might be disregarded from the analysis but detected genes will be unambiguously pathogen-associated genes).

In conclusion, we describe a 2-step protocol that provides for an enrichment of the original RNA sample followed by the biased linear amplification of pathogen transcripts that is potentially sufficient to accurately characterize the transcriptome of intracellular pathogens in *in vitro *or *in vivo *systems of infection.

## Competing interests

The authors declare that they have no competing interests.

## Authors' contributions

CAR conceived, designed and performed the experiments, and drafted the manuscript. CLG performed the computational analysis and drafted the manuscript. HRG helped to analyze the data and critically revised the manuscript. LGA conceived and coordinated the study and helped to draft the manuscript. All authors read and approved the final manuscript.

## Supplementary Material

Additional file 1 ***B. melitensis *16 M genome-directed primers (*Bm*GDPs)**. This table describes the minimal number (89) of primers to specifically anneal to the 3,198 *B. melitensis *ORFs. Primers were designed using a computer-based algorithm (GDP-Finder) that predicts reverse primers of 8-mer oligonucleotides required to anneal to all ORFs in a given genome. Click here for file
