# Supplementary material for: Selective amplification of Brucella melitensis mRNA from a mixed host-pathogen total RNA
Source: BMC Res Notes. 2010 Sep 28;3:244. doi: 10.1186/1756-0500-3-244 (PMC2954846; doi:10.1186/1756-0500-3-244)
Supplement: Additional file 1 — B. melitensis 16 M genome-directed primers (BmGDPs). This table describes the minimal number (89) of primers to specifically anneal to the 3,198 B. melitensis ORFs. Primers were designed using a computer-based algorithm (GDP-Finder) that predicts reverse primers of 8-mer oligonucleotides required to anneal to all ORFs in a given genome. [file 1756-0500-3-244-S1.DOC]

**Supplemental table S1. *B. melitensis* 16M genome-directed primers (*Bm*GDPs)**

| **Order** | **Primers** | **Unique # of ORFs covered** | **# ORFs covered** | **Percent complete** |
| --- | --- | --- | --- | --- |
| 1 | CGGCAAGC | 291 | 291 | 9.10 |
| 2 | CCAGCGCC | 248 | 539 | 16.85 |
| 3 | CGCCGCGC | 223 | 762 | 23.83 |
| 4 | CCTTGCCG | 201 | 963 | 30.11 |
| 5 | GCGCGCGC | 171 | 1134 | 35.46 |
| 6 | GCCGGAAA | 161 | 1295 | 40.49 |
| 7 | CGCCGCCG | 133 | 1428 | 44.65 |
| 8 | GGCGCGGC | 122 | 1550 | 48.47 |
| 9 | GCGCCAGC | 110 | 1660 | 51.90 |
| 10 | TTCCGGCA | 97 | 1757 | 54.94 |
| 11 | GCTTGCGC | 93 | 1850 |  |
| 12 | CGATCAGC | 85 | 1935 |  |
| 13 | GCCGCCAT | 76 | 2011 |  |
| 14 | TTCGGCAA | 70 | 2081 |  |
| 15 | CCTTGCGG | 68 | 2149 |  |
| 16 | CGATGATG | 64 | 2213 |  |
| 17 | CCGCGCCG | 56 | 2269 |  |
| 18 | CATCGGCA | 53 | 2322 |  |
| 19 | CGGCGGCA | 47 | 2369 |  |
| 20 | CCAGATCG | 43 | 2412 | 75.42 |
| 21 | GCTTGCCG | 40 | 2452 |  |
| 22 | CCAGAAGC | 40 | 2492 |  |
| 23 | GCGATGCG | 38 | 2530 |  |
| 24 | GCGCGCGG | 33 | 2563 |  |
| 25 | CCTTCGGC | 32 | 2595 |  |
| 26 | CATCGCGC | 30 | 2625 |  |
| 27 | CGCCTTCA | 28 | 2653 |  |
| 28 | TTCCAGCG | 27 | 2680 |  |
| 29 | CTTCCTTG | 27 | 2707 |  |
| 30 | AGGCCGAT | 25 | 2732 | 85.43 |
| 31 | CCATGCCG | 23 | 2755 |  |
| 32 | TTCCTGCG | 22 | 2777 |  |
| 33 | AATGCCGC | 20 | 2797 |  |
| 34 | GCGCGAAA | 17 | 2814 |  |
| 35 | CCATTGCG | 18 | 2832 |  |
| 36 | CCGCCAGC | 18 | 2850 |  |
| 37 | TTCGGAAA | 18 | 2868 |  |
| 38 | CAGCGCAT | 15 | 2883 |  |
| 39 | GCCTTTTC | 15 | 2898 |  |
| 40 | GGCGGAAA | 15 | 2913 | 91.09 |
| 41 | GATGCGGC | 14 | 2927 |  |
| 42 | GCCAAGCG | 13 | 2940 |  |
| 43 | GCCTGCGC | 12 | 2952 |  |
| 44 | CGGCATCG | 12 | 2964 |  |
| 45 | CGCCATCG | 10 | 2974 |  |
| 46 | GCCAGAAC | 11 | 2985 |  |
| 47 | TGAAGCGG | 11 | 2996 |  |
| 48 | GCACCAGC | 8 | 3004 |  |
| 49 | CGGCAGAT | 10 | 3014 |  |
| 50 | CCGCCTTC | 9 | 3023 | 94.53 |
| 51 | CTTGATGA | 9 | 3032 |  |
| 52 | AAACCGGA | 9 | 3041 |  |
| 53 | AAGCGGCA | 8 | 3049 |  |
| 54 | GCGGCGCC | 8 | 3057 |  |
| 55 | GCGCTCGC | 6 | 3063 |  |
| 56 | CCGCTTTC | 7 | 3070 |  |
| 57 | TCAATGGC | 7 | 3077 |  |
| 58 | TCTTCAAA | 7 | 3084 |  |
| 59 | ATGGCGGC | 5 | 3089 |  |
| 60 | GCCGCCAA | 6 | 3095 | 96.78 |
| 61 | TTTTCGCC | 6 | 3101 |  |
| 62 | GAAATCAA | 6 | 3107 |  |
| 63 | AAGCAAGG | 6 | 3113 |  |
| 64 | TTCGGCCA | 5 | 3118 |  |
| 65 | TTCATCGA | 5 | 3123 |  |
| 66 | GCCGAGAA | 3 | 3126 |  |
| 67 | GAAATCCG | 5 | 3131 |  |
| 68 | CCAATGCA | 5 | 3136 |  |
| 69 | GGCGGCGA | 3 | 3139 |  |
| 70 | CGGCGATG | 4 | 3143 | 98.28 |
| 71 | CGAGATCG | 4 | 3147 |  |
| 72 | TTGCGCAG | 4 | 3151 |  |
| 73 | AAGCCCGC | 4 | 3155 |  |
| 74 | TCACGCCG | 4 | 3159 |  |
| 75 | CGCAATAT | 4 | 3163 |  |
| 76 | AATGGAAA | 3 | 3166 |  |
| 77 | CATCGATG | 3 | 3169 |  |
| 78 | GCGACAGC | 3 | 3172 |  |
| 79 | CAGCCGGA | 3 | 3175 |  |
| 80 | CCATATCC | 3 | 3178 | 99.37 |
| 81 | CCCGCGCA | 3 | 3181 |  |
| 82 | TGCTCATC | 3 | 3184 |  |
| 83 | ACTGTTCC | 3 | 3187 |  |
| 84 | GATGATCG | 2 | 3189 |  |
| 85 | CGACCAGC | 2 | 3191 |  |
| 86 | TGATATCG | 2 | 3193 |  |
| 87 | AATTTCCG | 2 | 3195 |  |
| 88 | CGCAATAA | 2 | 3197 |  |
| 89 | GCATTGGC | 1 | 3198 | 100.00 |
